# A Unique Case of Primary Cutaneous Adenoid Cystic Carcinoma Associated with Aplasia Cutis Congenita in a Four-Year-Old Female: A Case Report

**DOI:** 10.3390/children9020292

**Published:** 2022-02-21

**Authors:** Andrea Zulli, Alessandra Martin, Flavio Facchini, Riccardo Coletta, Angela Tamburini, Teresa Oranges, Cesare Filippeschi, Andrea Bassi, Anna Maria Buccoliero, Antonino Morabito

**Affiliations:** 1 Department of Pediatric Surgery, Meyer Children’s Hospital, 50139 Florence, Italy; alessandra.martin@meyer.it (A.M.); flavio.facchini@meyer.it (F.F.); riccardo.coletta@meyer.it (R.C.); antonino.morabito@meyer.it (A.M.); 2 Department of Hematology-Oncology, Meyer Children’s Hospital, 50139 Florence, Italy; angela.tamburini@meyer.it; 3 Dermatology Unit, Department of Pediatrics, Meyer Children’s University Hospital, 50139 Florence, Italy; teresa.oranges@meyer.it (T.O.); cesare.filippeschi@meyer.it (C.F.); andrea.bassi@meyer.it (A.B.); 4 Pathology Unit, Meyer Children’s Hospital, 50139 Florence, Italy; annamaria.buccoliero@meyer.it

**Keywords:** children, primary cutaneous adenoid-cystic carcinoma, PCACC, aplasia cutis congenita, ACC

## Abstract

Introduction: Primary cutaneous adenoid-cystic carcinoma (PCACC) is a rare malignant tumour reported in only about 450 cases in the literature, with only two adolescent cases reported. PCACC seems to occur between the fifth and seventh decade of life, and the most frequent regions involved are head and neck (46%). Aplasia cutis congenita (ACC) has an incidence of 1:10,000, and it seems to be rarely associated with neoplastic lesions. Interestingly, the association between PCACC and ACC has, so far, never been described. Methods: We report a case of PCACC in the scalp associated with ACC in a four-year-old patient. Discussion: The patient was under follow-up at the dermatology unit, but suddenly a red lesion appeared within the ACC. This red, ulcerated area increased rapidly over six months, so it was surgically removed, and the pathological examination results were suggestive for cribriform PCACC. According to the guidelines for skin tumours, the patient underwent widening resection, and an advancement-sliding skin flap was performed to recreate the scalp. After one year of follow-up, the patient has no local or widespread recurrence of the PCACC, and the surgical scar appears to have healed well. Conclusions: This clinical case is the first known patient with PCACC associated with ACC. A skin excision biopsy should be performed with wide margins to avoid a second widening resection of skin in a similar scenario. Genetic studies may help to identify the origin of this rare association.

## 1. Introduction

Primary cutaneous adenoid cystic carcinoma (PCACC) is a rare, slow-growing malignancy tumour that usually affects middle-aged and older individuals. It frequently develops on the head, neck, chest or abdomen, and is characterised by aggressive local behaviour, with a very low incidence of metastasis [1,2,3,4,5,6,7,8].

Aplasia cutis congenital (ACC) is a rare skin disorder characterised by a localised absence of skin, usually located on the scalp. Still, it can occur anywhere on the body, including the face, trunk and extremities [9]. ACC may occasionally be associated with other anomalies such as syndromic pathologies. ACC has an incidence of 1:10,000, and it seems to be rarely associated with neoplastic lesions.

We describe a case of PCACC in a four-year-old female referred to our clinic for an aplasia cutis congenital of the scalp.

## 2. Methods

Case Report

**Figure 1:**
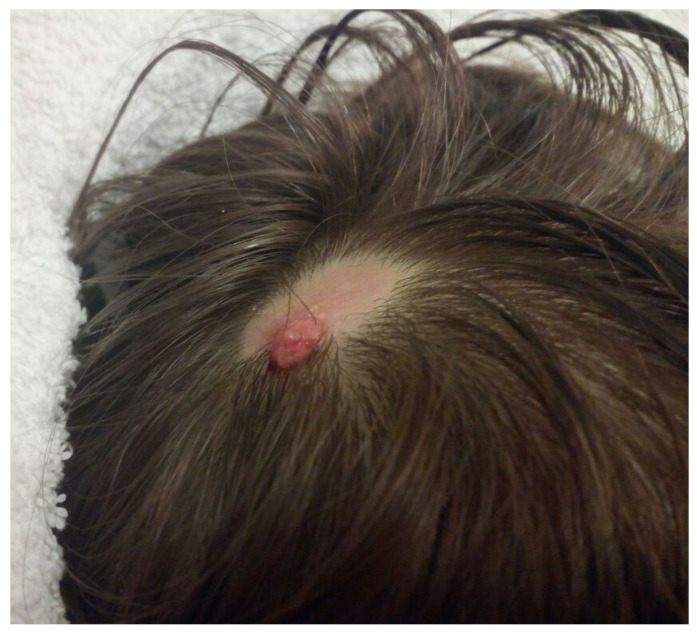
Small hyperemic granulomatous area on ACC.

The female patient has been under clinical follow-up at the dermatology unit since her neonatal period due to an ACC of the scalp. At the age of four, a small hyperemic granulomatous area (about 0.5 cm, Figure 1) appeared on the ACC, an indication for surgical excision.

**Figure 2:**
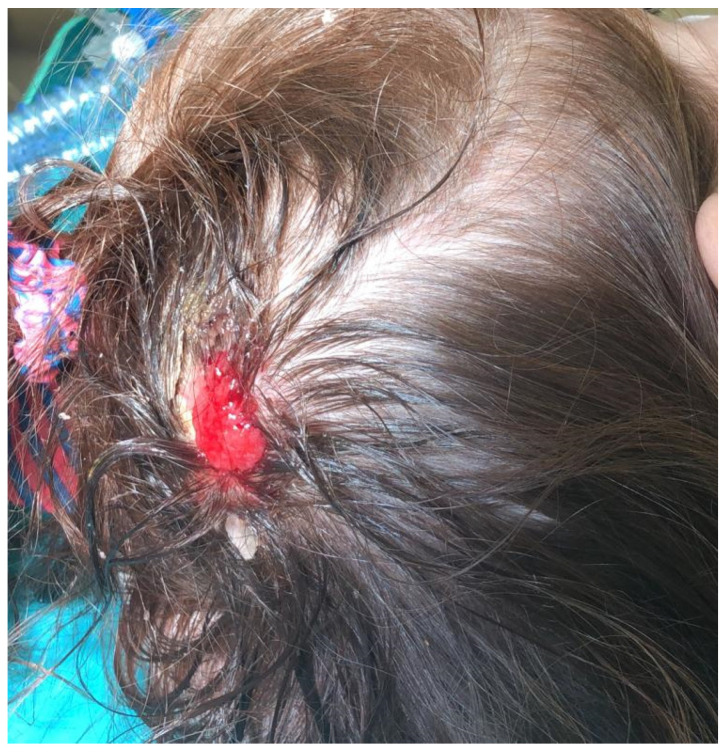
Areas of ulceration on the previous lesion.

During the waiting period for surgery, the lesion increased rapidly (from 0.5 to 1.5 cm) and showed areas of ulceration (Figure 2).

Under general anaesthesia, the lesion was surgically removed with the entire ACC area mainly involved; the excised tissue was sent for pathological examination. The postoperative course was regular, and the patient did not develop wound infection or any other complications.

**Figure 3:**
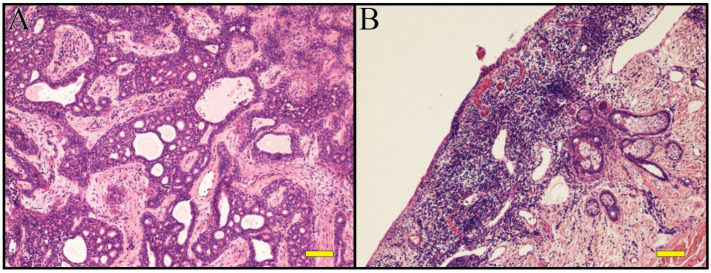
(**A**,**B**): Hematoxylin-Eosin. Primary cutaneous adenoid-cystic carcinoma on aplasia cutis of the scalp. Scale bar 100 µm.

The histological aspect of the lesion was suggestive of cribriform PCACC (Figure 3A,B), with Mib-1 (18%), AE1/AE3, CAM5.2, CEA, EMA, CD117 positivity.

According to the guidelines for this rare tumour [10], the patient underwent a second surgery less than one month later with the widening of the resection and advancement-sliding skin flaps to recreate the scalp.

The patient was referred to the oncology unit for clinical follow-up following the histological results.

**Figure 4:**
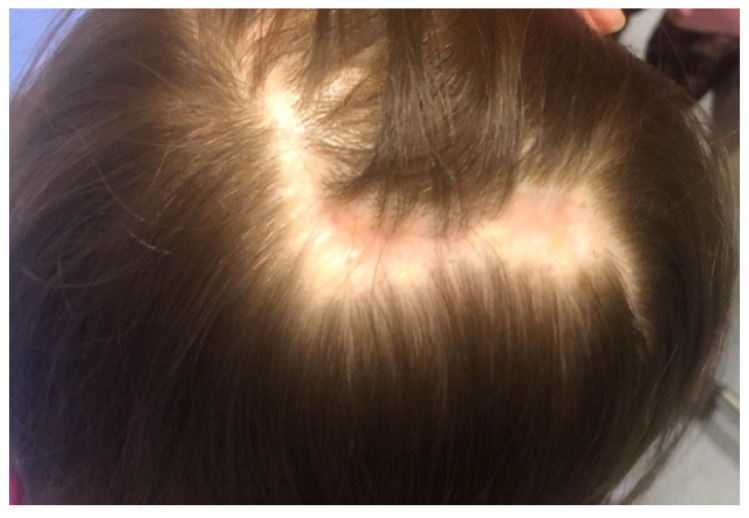
Well-healed surgical scar.

After six months of follow-up, the patient has no local or widespread recurrence of the PCACC, as determined by clinical examination and radiological exams (Chest X-ray and Abdomen-US). The surgical scar appears to have healed well (Figure 4).

## 3. Discussion

ACC is a congenital condition in which skin is absent, with the variable absence of underlying structures such as bone [9]. It most frequently affects the scalp, but any body location can be affected [11].

It generally presents as a solitary lesion, although there may be multiple locations on or near the vertex, with alopecia ranging from a few millimetres to more than 10 cm [9].

Most patients have no associated abnormalities, but some non-isolated ACC can cause gastrointestinal (29% intestinal fixation defects, 19% intestinal atresia and stenosis, 14% Meckel’s diverticulum), cardiac (33%), genitourinary (19%) (epispadias/hypospadias), musculoskeletal (19%), central nervous system (5%), and chromosome syndrome-associated diseases [12].

The cause of this condition is unclear and appears to be multifactorial.

ACC diagnosis is often clinical, followed by radiological examinations, and treatment usually involves a conservative approach to allow secondary intention healing (gentle cleansing, topical antiseptics, hydrocolloid dressings). Sometimes skin flaps and grafts can be used for more extensive and profound defects to prevent haemorrhage and infection [13].

PCACC is a rare malignant skin appendageal tumour first described by Boggio in 1975 [3].

Remarkrishnan et al. reported that PCACCs occur more frequently on the head and neck (46%), on the upper limbs (17%), trunk (15%), and lower limbs (13%). Reports of distant metastases were very low, with about 4% lymph node and 7% distant organ involvement [8].

Reviewing the literature, only about 450 cases of PCACC have been reported [1,2,3,4,5,6,7,14,15,16,17,18,19,20,21,22,23,24,25,26,27,28], with only two young patients (14 years of age) recorded [1].

The histogenesis of PCACC is still uncertain: an apocrine or eccrine derivation is debated [29]. Three major variant histologic growth patterns are described: cribriform, tubular and solid. The solid pattern is considered the more aggressive one [30].

Histological examination shows a tumour that consists of basophilic cells with a distinct adenoid or cribriform pattern in the mid to deep reticular dermis, with often myoepithelial cells surrounded by true lamina [14].

In his study, Alkan BI et al. reported that PCACC showed BerEp4, CEA, CD117(C-kit), and CK7 expression in the tumour periluminal regions, and p63 and SMA positivity in myoepithelial cells at the periphery of the cell islands [31]. Immunochemical analysis by Bergman et al. revealed intense staining with EMA and S100, and cytokeratin immunoperoxidase staining as generally positive. CEA monoclonal antibody positivity has also been reported [32].

Definitive diagnosis relies on histological and immune–histochemical characteristic features.

The standard treatment of PCACC described in the literature is wide surgical excision with at least a 2 cm safety margin from the tumour. Wide excision has the purpose of avoiding frequent recurrence [27].

In our case, the tumour was resected entirely after the first surgery, but a second surgery with the widening of the resection and advancement-sliding skin flaps to recreate the scalp was required after the histological close-margins report.

The benignity of the basal condition (ACC) made it difficult to hypothesise the onset of a malignant disease not previously reported. In the reported case report, the tumour showed an atypical behaviour compared to the cases previously reported in literature, although only two adolescent cases are reported in the literature [1]. Moreover, PCACC-associated alopecia has been reported in the literature when the scalp is involved [3], but ACC hid any evidence of this feature in this case.

A second surgery, widening the resection (more than 2 cm from the tumour margin) and using advancement-sliding skin flaps to recreate the scalp—a complex region to perform a primary closure of a wound—was performed.

No recurrence of the tumour was noted as of twelve months post-operation, with ongoing oncological follow-up.

## 4. Conclusions

We consider this an interesting case because ACC seems to be rarely predisposed to neoplastic lesions. Only two young patients (14 years of age) were described in the literature as being affected by PCACC. The association between PCACC and ACC has, so far, never been described.

Thus, we recommend that in a similar scenario, lesions appearing on ACC should not be underestimated, and a skin excision biopsy should be performed with wide margins to avoid a second widening resection of the skin.

A multidisciplinary approach with oncologists, paediatric surgeons, dermatologists, anatomopathologists, and other professional figures, remains of fundamental importance in improving these patients’ management and short- and long-term results.

## Data Availability

Not available.
